# Comparative Analysis of Panicker's Vacuum Suction Hemostatic Device Versus the Chhattisgarh Balloon Tamponade in the Management of Postpartum Hemorrhage at a Tertiary Health Facility in Uttar Pradesh, India

**DOI:** 10.7759/cureus.81399

**Published:** 2025-03-29

**Authors:** Preeti Tyagi, Anamika, Pratima Verma, Deepak Anand

**Affiliations:** 1 Obstetrics and Gynaecology, GSVM (Ganesh Shankar Vidyarthi Memorial) Medical College, Kanpur, IND; 2 Social and Preventive Medicine, GSVM (Ganesh Shankar Vidyarthi Memorial) Medical College, Kanpur, IND

**Keywords:** chhattisgarh balloon tamponade, hemostasis, panicker's suction cannula, post-partum hemorrhage, uterine atony

## Abstract

Introduction: Etiologies of postpartum hemorrhage vary widely and most commonly include uterine atony and placental site bleeding. The Chhattisgarh balloon tamponade applies positive pressure to the intrauterine myometrial wall and compresses the placental bed within the endometrial lining to stop postpartum hemorrhage. In contrast, Panicker's vacuum suction hemostatic device is used to perform vacuum retraction of the uterus. This study compared the effectiveness of Panicker’s vacuum suction hemostatic device and the Chhattisgarh balloon tamponade in controlling postpartum hemorrhage.

Methods: Patients older than 19 years of age with atonic postpartum hemorrhage were included in the study. Patients with other causes of postpartum hemorrhage, such as coagulopathy, traumatic postpartum hemorrhage, retained products of conception, uterine rupture, and perforation, were excluded. Patients were randomly divided into two groups (A and B), with 70 participants per group. In Groups A and B, Panicker's vacuum suction hemostatic device and the Chhattisgarh balloon tamponade were used, respectively. The outcome in both groups was measured based on different parameters.

Results: Statistically significant differences were found between Groups A and B regarding the time taken to control uterine bleeding, time taken to restore uterine tone, amount of blood loss, pain score, and length of hospital stay.

Conclusion: Vacuum retraction of the uterus using Panicker's vacuum suction hemostatic device was found to be more effective in the management of postpartum hemorrhage than the Chhattisgarh balloon tamponade.

## Introduction

Postpartum hemorrhage (PPH), defined as severe bleeding after childbirth, is the primary cause of maternal death worldwide. Globally, approximately 14 million women per year experience PPH, leading to 70,000 maternal deaths. Women who survive PPH may have permanent reproductive impairment and often require immediate surgical procedures to stop the bleeding [1]. In predominantly rural countries such as India, PPH occurs in approximately 12% of deliveries, with a high recurrence rate of 15% in subsequent pregnancies [2]. Postpartum hemorrhage (PPH) is defined by ACOG’s reVITALize program as cumulative blood loss ≥1000 mL or with signs and symptoms of hypovolemia within 24 hours of delivery [3]. The causes of PPH are diverse, with the most frequent being uterine atony and bleeding from the placental site (e.g., retained placenta or "placenta accreta spectrum"). Moreover, PPH can result from a large baby, prolonged labor leading to tissue injury, or underlying blood clotting disorders. Uterine atony is the leading cause of PPH in 80% of cases [4]. Conventional tamponade devices are used to stop the bleeding by applying positive pressure to the intrauterine myometrial wall and compressing the placental bed within the uterus [5]. Conservative management techniques such as uterotonic medications, which cause the uterus to contract, external uterine massage, and bimanual compression are generally used as “first-line” treatments [6]. The Chhattisgarh balloon tamponade is a uterine balloon tamponade with a central draining channel and is used for second-line management of atonic PPH [7].

A more recent development in the treatment of atonic PPH is vacuum retraction of the uterus, which facilitates uterine contraction and retraction. All bleeding arterioles and sinusoids get sucked in, which stops the bleeding owing to the negative pressure formed inside the uterine cavity. Devices used to perform vacuum retraction include Panicker's vacuum suction hemostatic device, SR suction cannula, suction tube uterine tamponade, and the Jada system [8].

Panicker’s vacuum suction hemostatic device is a steel or plastic tube (12 mm in diameter and 25 cm in length) with numerous perforations (4 mm in diameter) on the distal portion (12 cm) of the cannula [9].

India is a developing country with limited resources, and it is often challenging to control unexpected, severe bleeding owing to a lack of doctors and paramedics, a shortage of blood and blood products, and the need for transportation to more advanced medical facilities. There is no previous study comparing the effectiveness of Panicker’s vacuum suction hemostatic device and the Chhattisgarh balloon tamponade. Based on our observations prior to this study, it was expected that Panicker's vacuum suction hemostatic device would provide better results in controlling PPH. To confirm this observation, we carried out the study to determine the better option.

## Materials and methods

This study was performed at the Obstetrics and Gynecology Department of Ganesh Shankar Vidyarthi Memorial (GSVM) Medical College in Kanpur, Uttar Pradesh, India, between April 2022 and June 2024. The study aimed to compare the efficacy of two interventions, Panicker's hemostatic device and the Chhattisgarh balloon tamponade, in reducing postpartum hemorrhage. Study participants were randomly assigned to one of two groups (Group A and Group B), with 70 participants per group.

The study included women with atonic postpartum hemorrhage who were 19 years of age or older. Women who exhibited disseminated intravascular coagulation, had inherited blood clotting disorders, or had traumatic postpartum hemorrhage were excluded. Additionally, women with uterine rupture requiring cesarean hysterectomy, those with uterine perforations, and those experiencing secondary postpartum hemorrhage due to retained products of conception or placental anomalies were excluded.

Panicker's vacuum suction hemostatic device was used in Group A. The cannula was placed in the uterine cavity up to the fundus. It was inserted in the lithotomy position through the cervix in the case of vaginal delivery, and through the uterine wound in the case of lower segment cesarean section.

A negative suction pressure of 650-700 mm Hg was created within one minute and maintained for 10 to 15 minutes using a suction machine. This resulted in uterine retraction, and all blood accumulated in the uterine cavity was aspirated. During this process, soft cervical tissues were drawn into the uterine and cervical perforations of the cannula. The time required to achieve hemostasis was recorded. The cannula was reinserted if bleeding continued. This process was repeated every three hours or whenever bleeding resumed.

**Figure 1:**
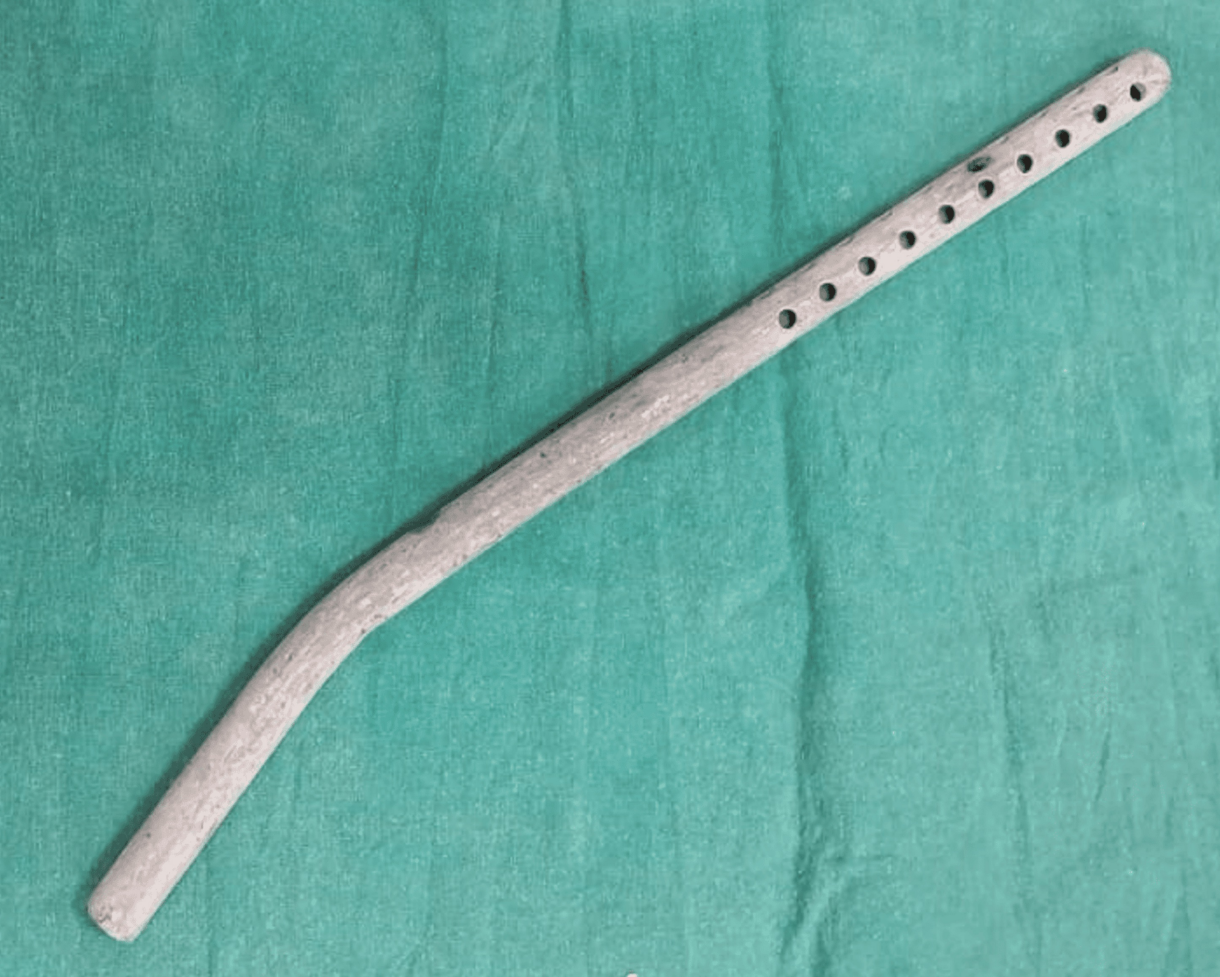
Panicker's vacuum hemostatic suction device without suction attachments.

**Figure 2:**
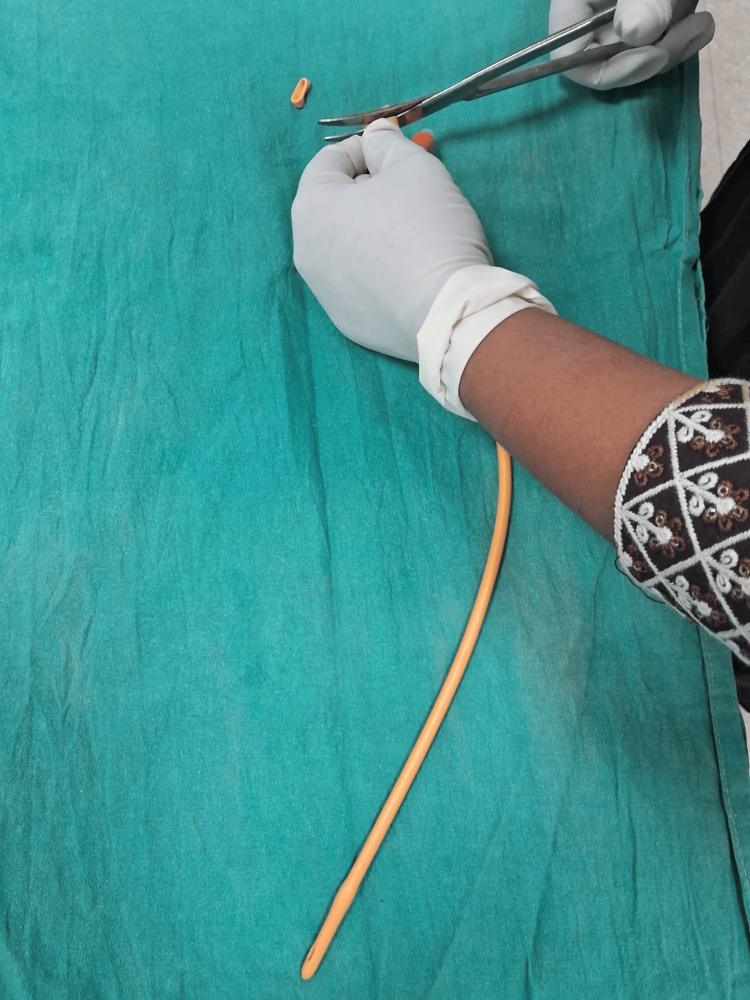
Drainage tube of a Foley catheter is cut into two ring-shaped segments.

**Figure 3:**
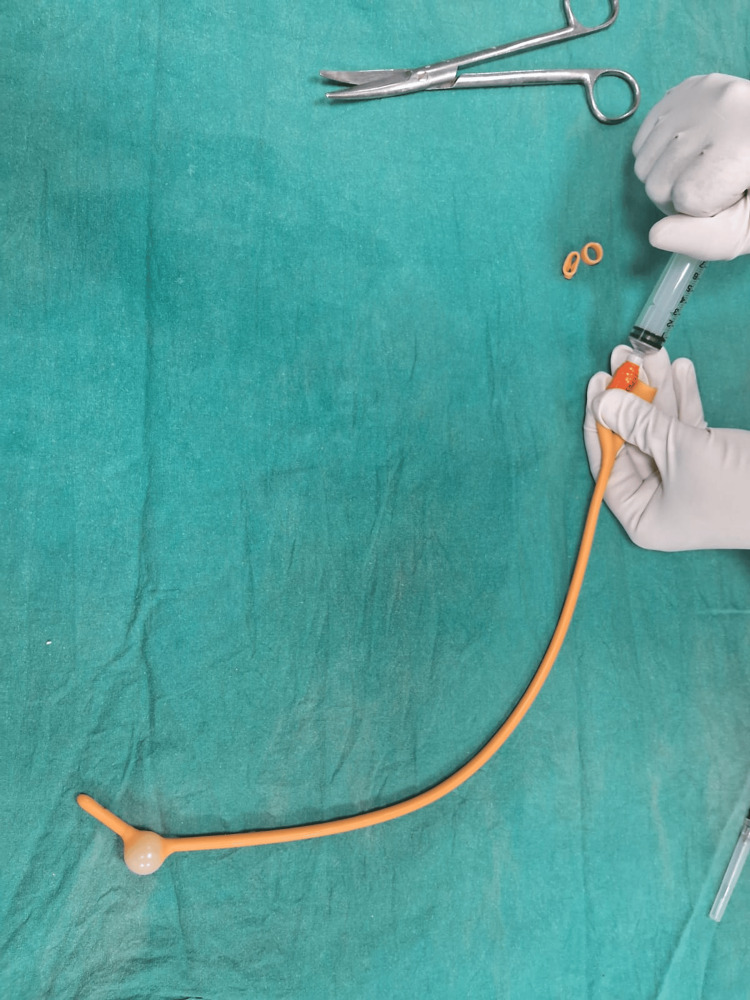
Bulb of the catheter is inflated by pumping 2-5 mL of air.

**Figure 4:**
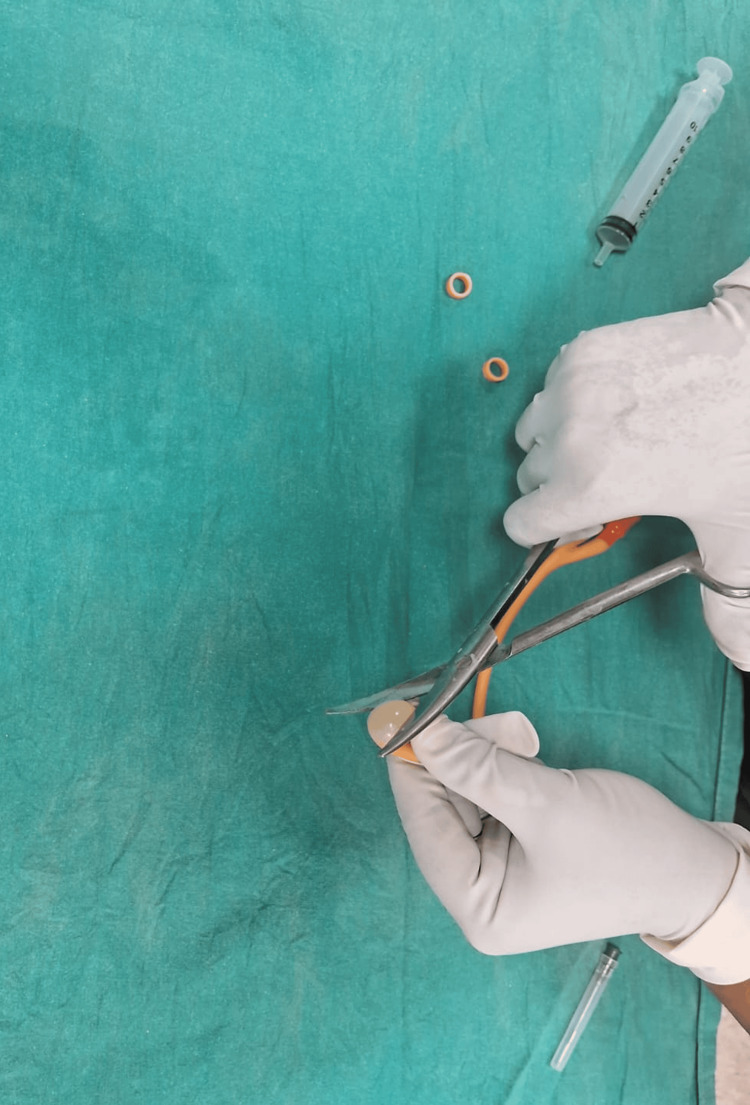
Bulb of the catheter is inflated by pumping 2-5 mL of air into it and then excised.

**Figure 5:**
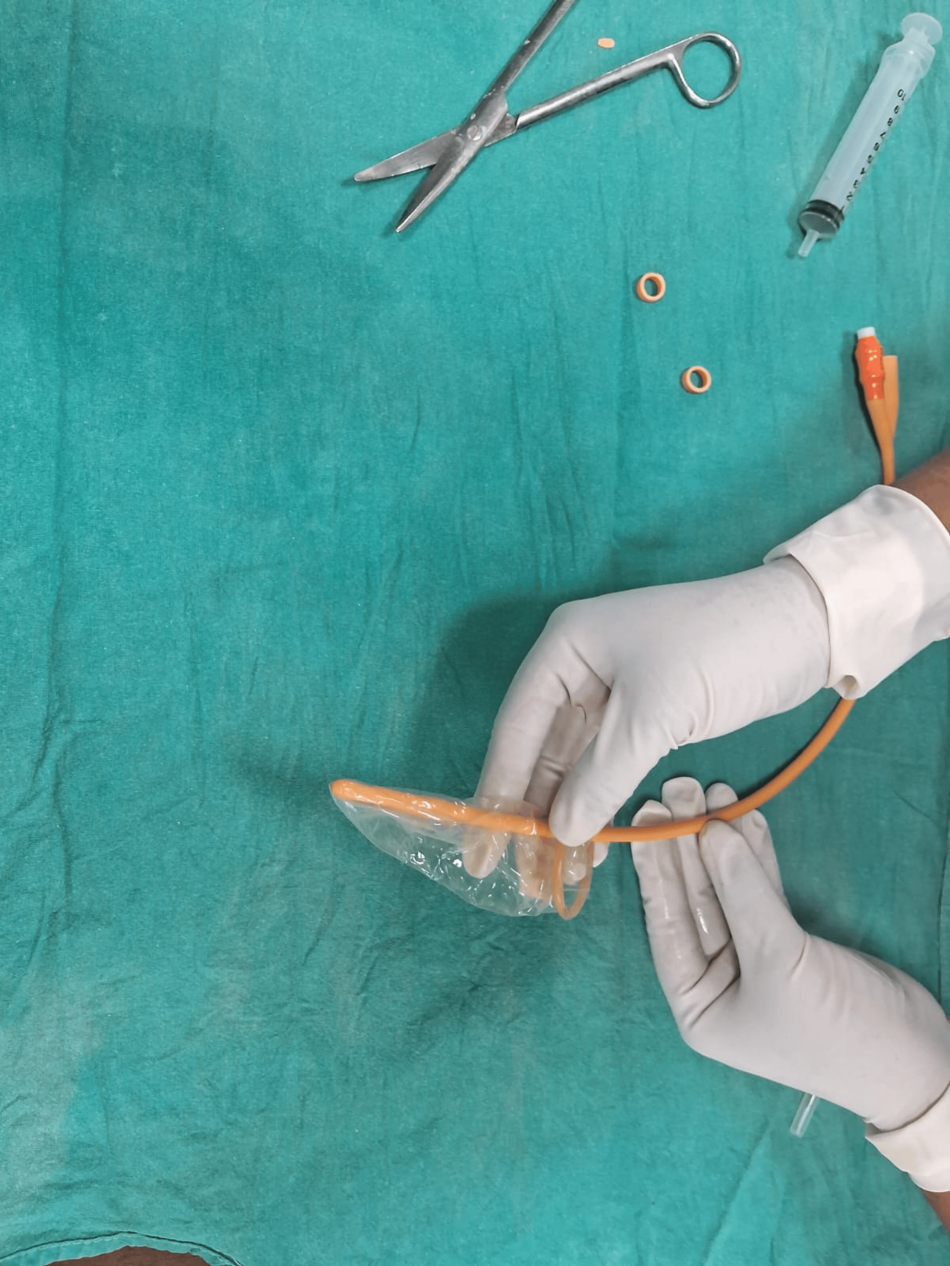
A condom is rolled over the catheter.

**Figure 6:**
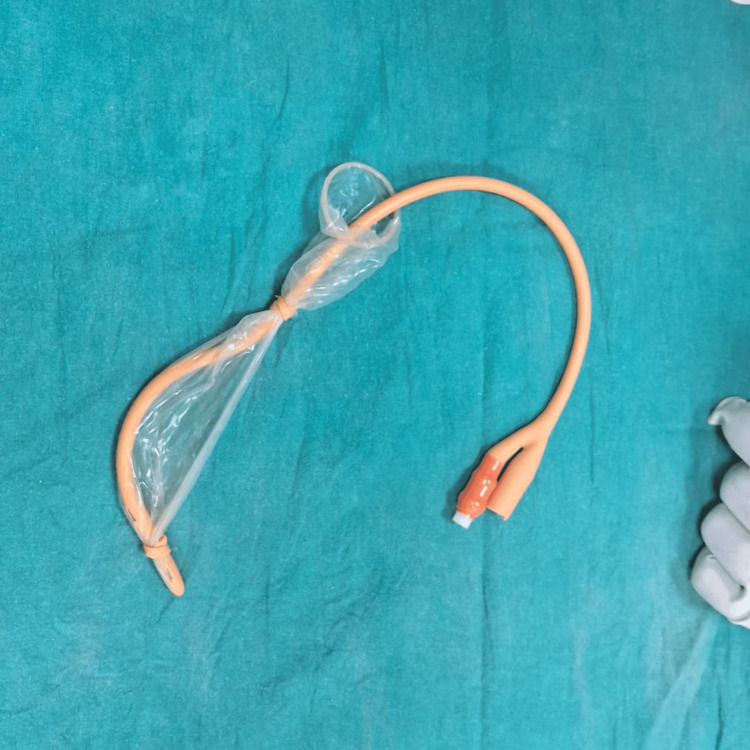
The rings are wrapped around the condom twice, leaving 1.5-2 cm of condom at either end.

**Figure 7:**
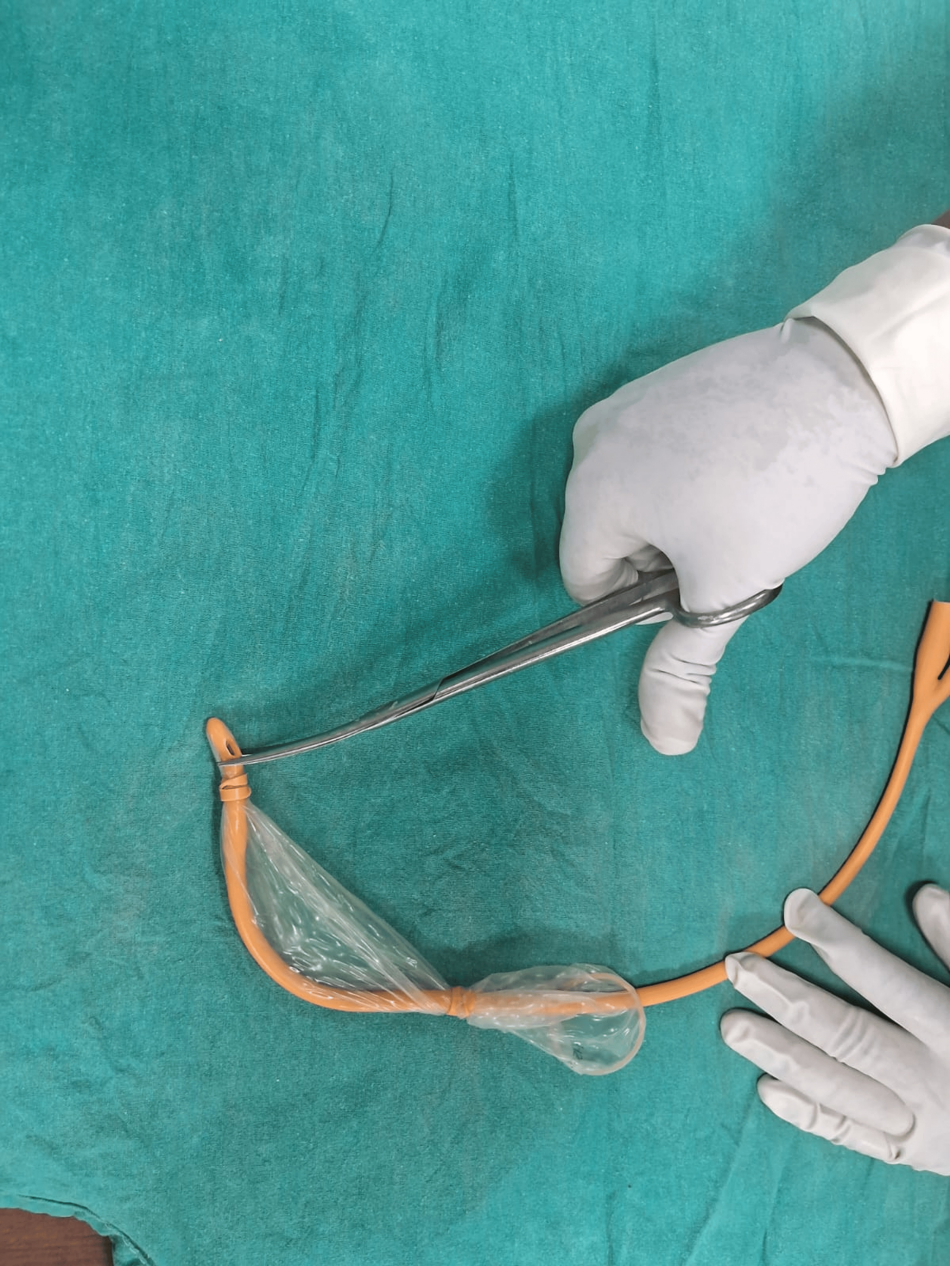
The tip of the catheter and the closed end of the condom are excised at approximately 0.5 cm from the tied ring, leaving both rings in place.

**Figure 8:**
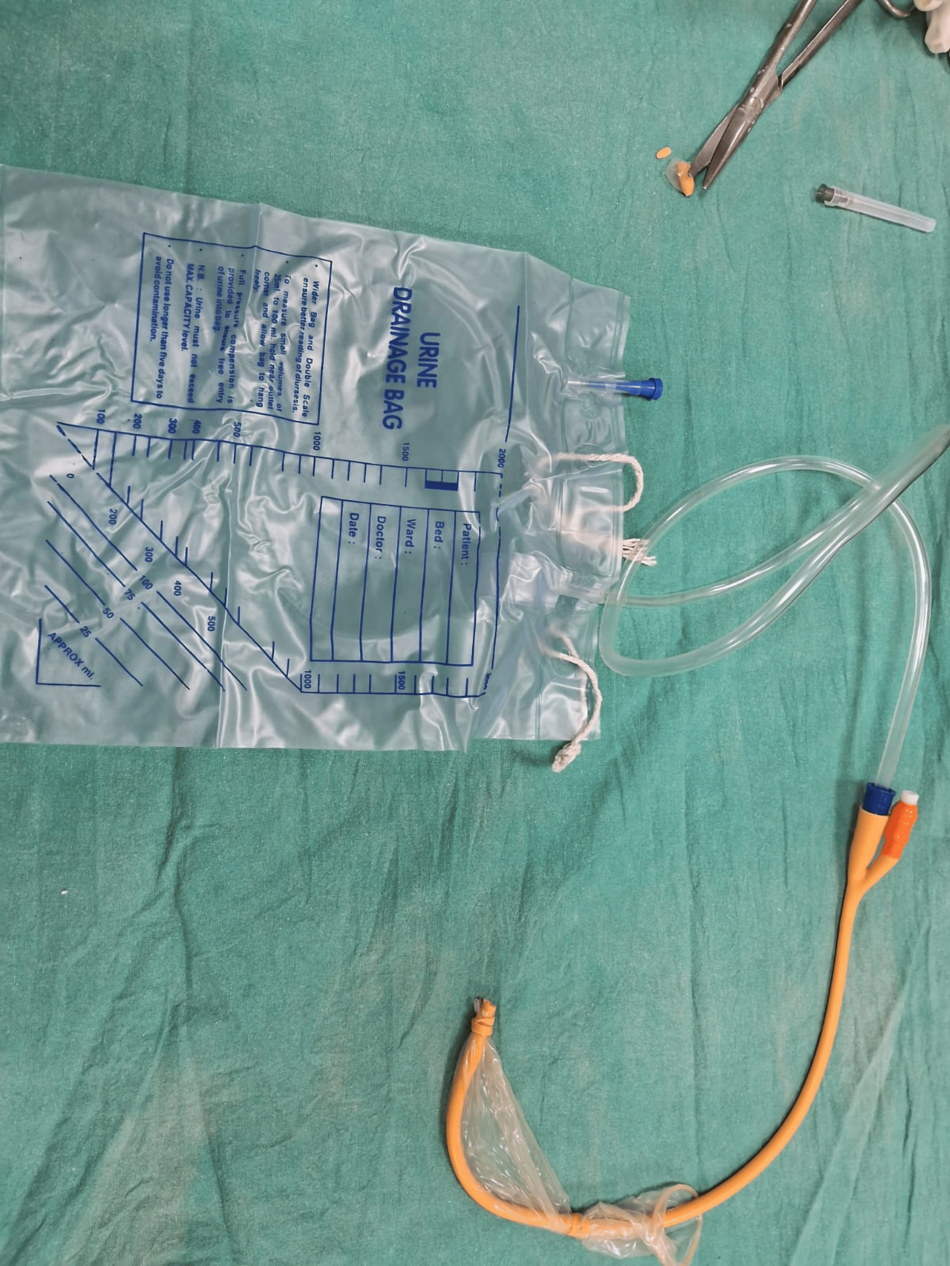
A drainage port was attached to a Urobag.

**Figure 9:**
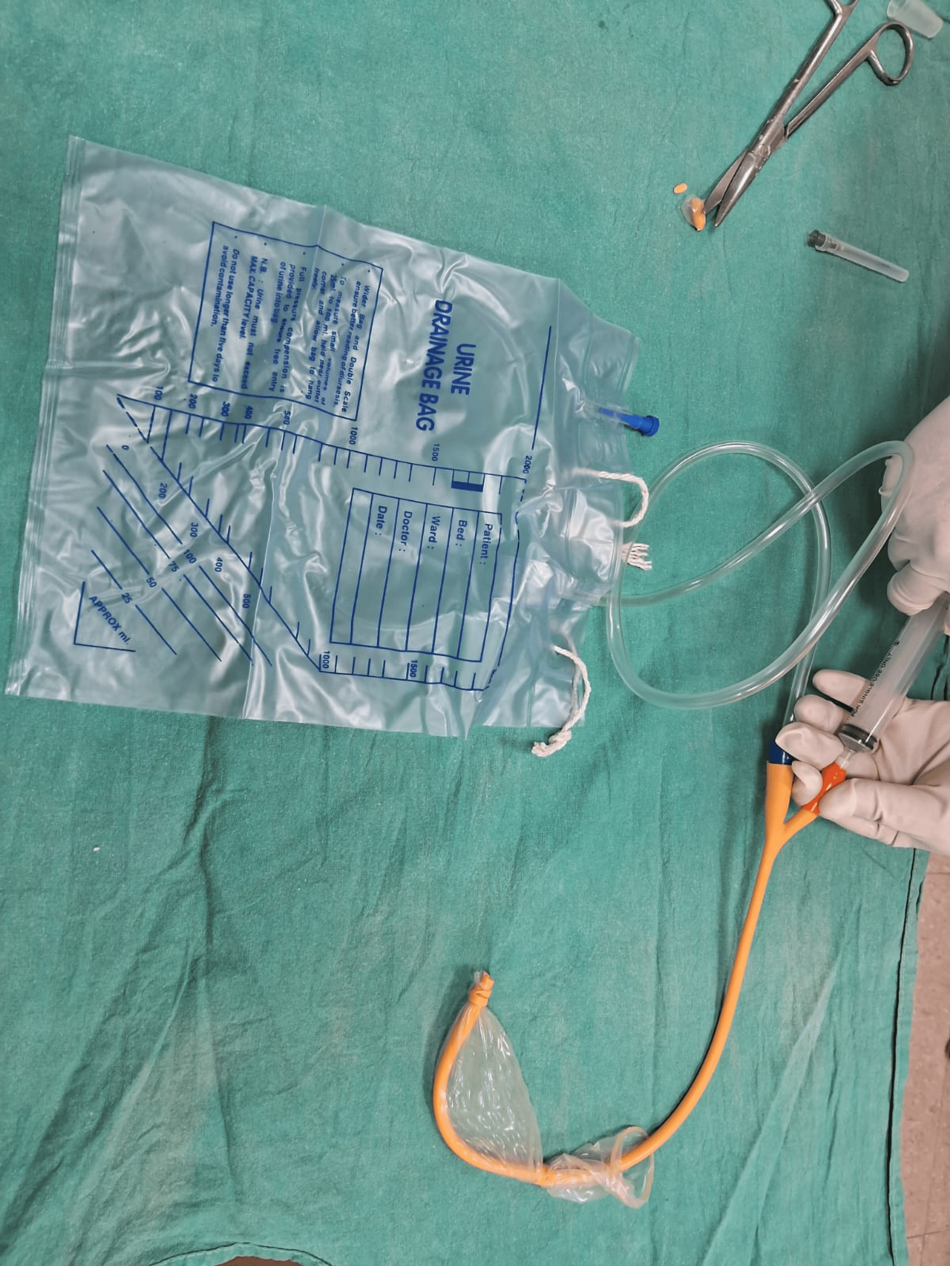
A balloon inflation port was used to inflate the balloon.

The Chhattisgarh balloon tamponade method was applied in Group B. The following steps were followed to prepare the Chhattisgarh balloon tamponade. First, the drainage tube of a Foley catheter was cut into two ring-shaped segments. Next, the bulb of the catheter was inflated by pumping 2 to 5 mL of air into it and then excised. A condom was then rolled over the catheter and secured at both ends using the two rings. The rings were wrapped around the condom twice, leaving 1.5 to 2 cm of the condom at either end. The tip of the catheter and the closed end of the condom were excised at approximately 0.5 cm from the tied ring, leaving both rings in place. A drainage port was attached to a Urobag. The balloon inflation port was used to inflate the balloon. In cases where the tamponade was evacuated during inflation, it was deflated, reinserted, and secured in place with vaginal packing before re-inflation. The process is illustrated using photographs (Figures 2-9).

Through this comparison, the study sought to determine which of the two devices, the Chhattisgarh balloon tamponade or Panicker's hemostatic device, offers better postpartum hemorrhage control.

To ensure consistency in practices and adherence to guidelines, all researchers and personnel responsible for data collection received training in the preparation and application of the Chhattisgarh balloon tamponade as well as in the use of Panicker's vacuum hemostatic suction device. They were also trained in the assessment of blood loss during PPH, maintaining records of the time taken to control the bleeding, and using the Universal Pain Assessment Tool. The degree of pain was assessed using the Universal Pain Assessment Tool [10], and the participants' socioeconomic status was assessed according to the modified Kuppuswamy socioeconomic scale 2024 [11]. The collected data were analyzed using IBM SPSS Statistics for Windows, Version 23 (Released 2015; IBM Corp., Armonk, New York).

## Results

Most participants in both groups were younger than 30 years of age, with 60 participants (85.71%) in Group A and 56 participants (80.00%) in Group B. According to the modified Kuppuswamy socioeconomic scale 2024, the participants were categorized into upper-middle, lower-middle, or lower socioeconomic classes. The largest proportion of participants, i.e., 37 (52.86%) in Group A and 34 (48.57%) in Group B, belonged to the lower-middle class. In terms of educational background, the highest percentage of participants in each group had completed formal education ranging from Class 6 to Class 12 (Table 1).

**Table 1:** Association between group and gravida status, mode of delivery and high-risk factors. Chi-square test; results are significant at p < 0.05.

Parameter	Group A, n (%)	Group B, n (%)	p-value
Gravida status			
≤2	36 (51.43%)	37 (52.86%)	0.866
≥3	34 (48.57%)	33 (47.14%)	-
Mode of delivery			
Vaginal	60 (85.71%)	33 (47.14%)	<0.001
Cesarean	10 (14.29%)	37 (52.86%)	
High-risk factor			
None	57 (81.43%)	57 (81.43%)	
Anemia	5 (7.14%)	3 (4.23%)	-
Hypothyroidism	4 (5.71%)	5 (7.14%)	0.936
Previous cesarean delivery	2 (2.86%)	2 (2.86%)	-
Diabetes	2 (2.86%)	3 (4.23%)	-

The gravida status of participants in Groups A and B was not significantly different. In terms of delivery method, the percentage of women who had a vaginal delivery was significantly higher in Group A than in Group B. However, when assessing the presence of high-risk factors, no significant difference was observed between the two groups (Table 2).

**Table 2:** Sociodemographic characteristics of the patients.

Variable	Group A, n (%)	Group B, n (%)
Age		
<30 years	60 (85.71%)	56 (80.00%)
≥30 years	10 (14.29%)	14 (20.00%)
Socioeconomic class		
Upper middle	9 (12.86%)	8 (11.43%)
Lower middle	37 (52.86%)	34 (48.57%)
Lower	24 (34.29%)	28 (40.00%)
Education level		
Uneducated	19 (27.14%)	11 (15.71%)
Classes 1–5	4 (5.71%)	7 (10.00%)
Classes 6–12	45 (64.29%)	48 (68.57%)
Graduate and postgraduate	2 (2.86%)	4 (5.71%)

The time required to stop uterine bleeding was significantly shorter in Group A. Moreover, these patients regained uterine tone in a significantly shorter period and experienced significantly lower total blood loss. However, no statistically significant differences were observed between Groups A and B in terms of pre-delivery and post-delivery hemoglobin levels, nor in the decline in hemoglobin levels associated with the procedure. Notably, participants in Group A reported significantly lower pain scores and had a significantly shorter hospital stay than those in Group B (Table 3).

**Table 3:** A comparison of the efficacy of Panicker's vacuum suction hemostatic device and Chhattisgarh balloon tamponade. Wilcoxon-Mann-Whitney U Test results are significant at p < 0.05.

Parameter	Group A (mean ± SD)	Group B (mean ± SD)	p-value
Time taken to stop the bleeding (min)	4.85 ± 0.89	8.61 ± 0.65	<0.0011
Time taken to restore uterine tone (min)	3.27 ± 0.81	6.72 ± 0.87	<0.0011
Amount of blood loss (mL)	178.80 ± 26.71	229.42 ± 20.21	<0.0011
Hemoglobin (g/dL, before delivery)	10.32 ± 1.53	10.31 ± 1.35	0.9671
Hemoglobin (g/dL, after delivery)	9.04 ± 1.23	8.91 ± 1.35	0.5531
Decrease in hemoglobin (g/dL)	1.28 ± 0.82	1.40 ± 0.50	0.2981
Pain score	2.62 ± 1.20	3.78 ± 1.40	<0.0011
Duration of hospital stay (days)	3.14 ± 1.10	5.13 ± 1.10	<0.0011

## Discussion

In India, there are limited resources in health facilities for the management of PPH. Both Panicker’s suction cannula and the Chhattisgarh balloon tamponade are promising modalities for the control of PPH. To identify the better option among them, we conducted the present study and compared the Chhattisgarh balloon tamponade with Panicker’s vacuum suction hemostatic device. In our study, it was found that the time required to stop the bleeding and the amount of blood loss were significantly less in Group A. These results are consistent with previous findings, which emphasize the superior efficacy of Panicker’s vacuum suction hemostatic device in the management of atonic PPH.

In 2021, Rana et al. conducted a study in patients with postpartum hemorrhage using Panicker’s cannula at M. K. Shah Medical College and Research Center, Ahmedabad (India). A total of 20 women with singleton pregnancies were included; of these, 14 had vaginal deliveries and 6 had lower-segment cesarean sections. With the use of Panicker’s suction cannula, complete cessation of bleeding, accompanied by contraction and firm retraction of the uterus, was achieved in all women within four minutes. The amount of blood collected in the suction apparatus ranged from 150 to 250 mL. This study demonstrated that vacuum retraction of the uterus supports the natural physiological processes of contraction and retraction [12].

In 2022, Sowjanya and Suseela conducted a study at the Government Medical College, Kadapa (India), to evaluate the efficacy of the SR suction cannula in preventing postpartum hemorrhage. It was found that the suction cannula successfully stopped bleeding within four minutes, with 100 to 200 mL of blood loss. The mean blood loss in the SR suction cannula group (187.6 mL) was significantly lower than that in the control group (378 mL). This finding is similar to ours and confirms that the suction procedure is a practical approach for controlling postpartum hemorrhage in resource-limited settings [13].

Likewise, Meena et al. highlighted that vacuum retraction of the uterus represents a novel advancement in managing atonic postpartum hemorrhage. Their findings indicated that generating negative pressure in the uterine cavity facilitates a reduction in uterine size, which supports the physiological process of uterine contraction and retraction to prevent atonic postpartum hemorrhage. Complete cessation of bleeding, accompanied by contraction and firm retraction of the uterus, was mostly observed within four minutes after the procedure was started [14]. However, a study by Mishra et al. using the Chhattisgarh balloon tamponade reported that the time taken from the diagnosis of atonic postpartum hemorrhage to the insertion of the Chhattisgarh balloon tamponade was 20 minutes. The amount of blood loss before insertion of the tamponade was approximately 1600 mL, and the mean blood loss after control of PPH was 35 mL in the subsequent 12 hours [7]. Samartha et al. also reported that vacuum retraction of the uterus leads to cessation of bleeding, associated with contraction and firm retraction of the uterus within four minutes [8]. Makhija et al. found that suction and evacuation are effective techniques, involving the maintenance of negative suction pressure in the uterine cavity, and they successfully stopped bleeding in 88.9% of patients [15]. Panicker analyzed the effectiveness of Panicker’s vacuum suction hemostatic device in the management of atonic postpartum hemorrhage in 40 women who had vaginal deliveries and 15 who had lower-segment cesarean sections at a low-resource maternity hospital over two years. After insertion of the cannula and maintenance of negative pressure at 650-700 mm Hg for 30 minutes, firm retraction of the uterus was observed, with blood loss ranging from 50 to 300 mL [9].

Limitations

The present study included only 140 participants (70 in each group), which may restrict the generalizability of the findings. Moreover, the results may not be representative of outcomes in other healthcare settings, particularly in rural or resource-constrained environments, as the study was limited to a single tertiary care facility. The findings may not be applicable to all occurrences of postpartum hemorrhage, as the study did not include patients with secondary postpartum hemorrhage, genetic blood coagulation disorders, or other complications. Additionally, the study focused primarily on short-term management and outcomes, rather than long-term impacts such as future fertility or healing. Although Panicker's device is affordable, we could not provide a thorough analysis comparing the costs associated with the two approaches. As both devices require clinical knowledge for insertion and management, differences in operator skill may lead to heterogeneity in outcomes. Lastly, our study did not fully document patient satisfaction, pain thresholds, or comfort with either device.

## Conclusions

The present findings suggest that Panicker’s vacuum suction hemostatic device is more user-friendly, requires less time and specialized training to operate, and is both inexpensive and reusable. Vacuum retraction of the uterus is a highly effective physical method that supports the natural physiological processes of uterine contraction and retraction to manage atonic postpartum hemorrhage. Additionally, the affordability and reusability of Panicker’s device can help alleviate the financial burden in developing countries with limited resources and financial constraints, such as India. In health centers where Panicker’s vacuum suction hemostatic device is unavailable, the Chhattisgarh balloon tamponade can be prepared and used.
